# The Sale of Poisons

**Published:** 1899-12-23

**Authors:** 


### THE SALE OF POISONS.

A curious and very important case came before the Lord Mayor at the Mansion House recently, when certain wholesale seed merchants were summoned for having contravened the provisions of the Pharmacy Act, 1868, in the sale of a certain vegetable poison called "nicotine." The substance in question, which had a special trade name of its own, was a composition to be vaporised in greenhouses for the destruction of insect life on plants. Dr. Thomas Stevenson, analyst to the Home Office, gave evidence that it was a solution of nicotine and camphor in diluted alcohol. It contained 37·3 per cent. of the alkaloid nicotine, 34·5 per cent. of camphor, and 15·1 per cent. of alcohol, the balance being water. A small drop from the bottle killed a rabbit in two and a half minutes, and the whole bottle contained enough to kill thousands of people, if swallowed. In reply to a question by the Lord Mayor, he said that three, four, or five drops of the preparation would be fatal to human life. The defence seemed to be that the substance was not pure nicotine, but a preparation of nicotine; and that preparations of this vegetable alkaloid were not scheduled. Fines were imposed, but a case was stated. The question raised is a most important one, and, from a legal point of view, may be open to argument; but, so far as the public is concerned, it seems a most serious matter that, while all sorts of difficulties are placed in the way of buying the ordinary poisons, such stuff as this, a bottleful of which would, according to the report in the *Times*, kill "thousands of people," should be sold without restriction. What is the law of the matter the lawyers must decide; but if, notwithstanding the Sale of Poisons Acts, such poisons as this can still be freely sold, one can hardly doubt that the law will have to be altered.

